# When it is not sacroiliitis

**DOI:** 10.1007/s00256-025-04958-7

**Published:** 2025-06-03

**Authors:** Anthony De Leeuw, Reda Cherkaoui Jaouad, Mohamed Kamoun, Souhir Abidi, Paul Michelin, Anne Cotten

**Affiliations:** 1 Department of Musculoskeletal Imaging, University of Lille, CHU Lille, 59000 Lille, France; 2 Department of Musculoskeletal Imaging, University of Rouen, CHU Rouen, 76000 Rouen, France; 3 MABLab-Marrow Adiposity and Bone Lab ULR 4490, University of Lille, 59000 Lille, France

**Keywords:** MRI, Osteoarthritis, Sacroiliac joint, Sacroiliitis, Spondyloarthritis

## Abstract

Magnetic resonance imaging of the sacroiliac joints (SIJ) is now frequently performed to detect subchondral inflammatory and structural changes in patients with early axial spondyloarthritis (SpA). However, similar changes can also occur in various other conditions, which may lead to the overdiagnosis of axial SpA. The aim of this article is to review the key imaging features of the most common disorders that may mimic inflammatory sacroiliitis, including mechanical changes and osteoarthritis, osteitis condensans ilii and pregnancy-related changes, other strain related changes, anatomical variants, pediatric SIJs, hyperostosis, infectious sacroiliitis, SAPHO syndrome, hyperparathyroidism, and sacral stress fractures.

## Introduction

Magnetic resonance imaging (MRI) of the sacroiliac joints (SIJ) is now frequently performed to identify patients with early axial spondyloarthritis (axSpa) before the development of the radiographic features of sacroiliitis [1]. The presence of subchondral bone marrow edema on fluid-sensitive MR sequences is indicative of active sacroiliitis [1, 2]. Moreover, MRI can demonstrate additional inflammatory features, such as enthesitis and capsulitis, as well as structural changes, including erosion, fat metaplasia, sclerosis, and ankylosis [3, 4]. However, various other disorders can also be associated with inflammatory or structural changes of the SIJs, potentially leading to the overdiagnosis of axial SpA and exposing the patients to costly and unnecessary treatments with unjustifiable side effects [5]. Consequently, careful interpretation of SIJ MRI is essential.

The aim of this article is to review the most frequent differential diagnoses that may mimic inflammatory sacroiliitis in clinical practice.

## Mechanical changes and osteoarthritis

The SIJ is a complex and unique joint composed of an anterior/inferior cartilaginous compartment and a posterior/superior ligamentous compartment [6, 7]. Its primary function is to transmit loads between the axial skeleton and the lower limbs [7]. As a result, a considerable strain is put on the SIJ, which may lead to early mechanical and degenerative changes. While these changes are often asymptomatic, some may be associated with pain [8]. They are more commonly observed in women, possibly due to a smaller load-bearing cartilaginous surface and a more horizontally oriented sacrum [7, 9]. Although these changes are frequently seen in middle-aged and elderly individuals, they can be detected as early as in the 20 s [9]. It is estimated that mechanical SIJ diseases are 20 to 100 times more prevalent than inflammatory sacroiliitis [10].

**Fig. 1:**
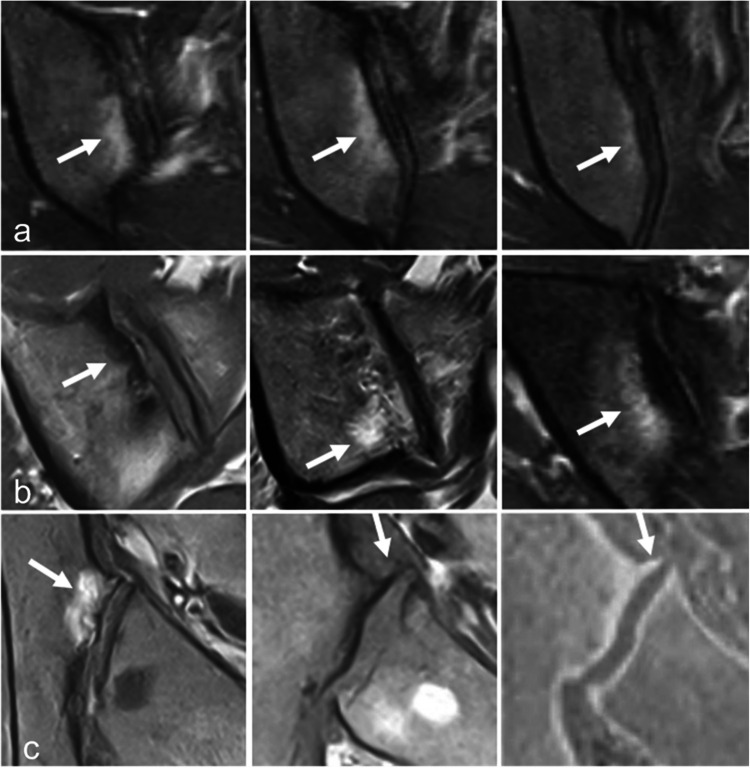
Mechanical changes at the anterior aspect of the middle third of a right SIJ. **a** Edematous changes in the iliac bone (arrow) on three contiguous coronal oblique T2-weighted images in a 41-year-old woman. **b** Sclerotic, fatty, and mixed subchondral changes (arrows) on coronal oblique T1-, T1-, and fat-saturated (FS) T2-weighted images in three different patients, respectively. **c** Subchondral bone marrow edema (arrow) on an axial oblique FS T2-weighted image and osteophytes (arrow) on T1- and StarVIBE T1-weighted images in the same patient as in **a**

Mechanical and degenerative subchondral changes represent the main pitfall in the interpretation of SIJ MRI, as they can manifest as subchondral bone marrow edema (BME), sclerosis, fat deposition, or associated features (Fig. 1) [11]. However, they are typically located in the anterior part of the middle third of the SIJ, which is the area where mechanical stress is concentrated [11–13]. Due to the C shaped morphology of the cartilaginous component of the SIJ, these focal changes are depicted on the most anterior coronal oblique sections oriented along the S1–S2 axis, often predominating on the iliac side (Fig. 1) [5, 6, 11]. Axial orthogonal oblique images easily confirm the anterior location of these mechanical changes (Fig. 1), which are typically triangular in shape and bilateral [11]. These changes are well-demarcated, limited in size, without significant erosion. Additional features, such as osteophytes, joint space narrowing, and subchondral cysts, may be more challenging to detect by MRI [9, 12, 14], although CT-like images may enhance their visualization (Fig. 1) [15]. Importantly, there is no involvement of the remaining SIJ [11, 13].

## Osteitis condensans ilii and pregnancy-related changes

Osteitis condensans ilii (OCI) was initially described on pelvic X-rays as a unilateral or, more commonly, bilateral triangular-shaped sclerosis in the ilium corresponding to the weight-bearing portion of the SIJ. Extensive mechanical stress on the SIJ is usually considered the underlying cause [16–19]. This sclerosis is typically dense, compact, homogeneous, well-delineated, without significant erosive changes. Although more extensive than the mechanical changes described above, it typically predominates in the anterior part of the middle third of the SIJ [11]. The prevalence of OCI is higher in postpartum women, but it can also occur in nulliparous women and in men, and may be seen in association with increased pelvic tilt, scoliosis, and obesity [20–22]. It is usually asymptomatic and often found incidentally on imaging, although it can be less commonly, associated with low back pain.

**Fig. 2:**
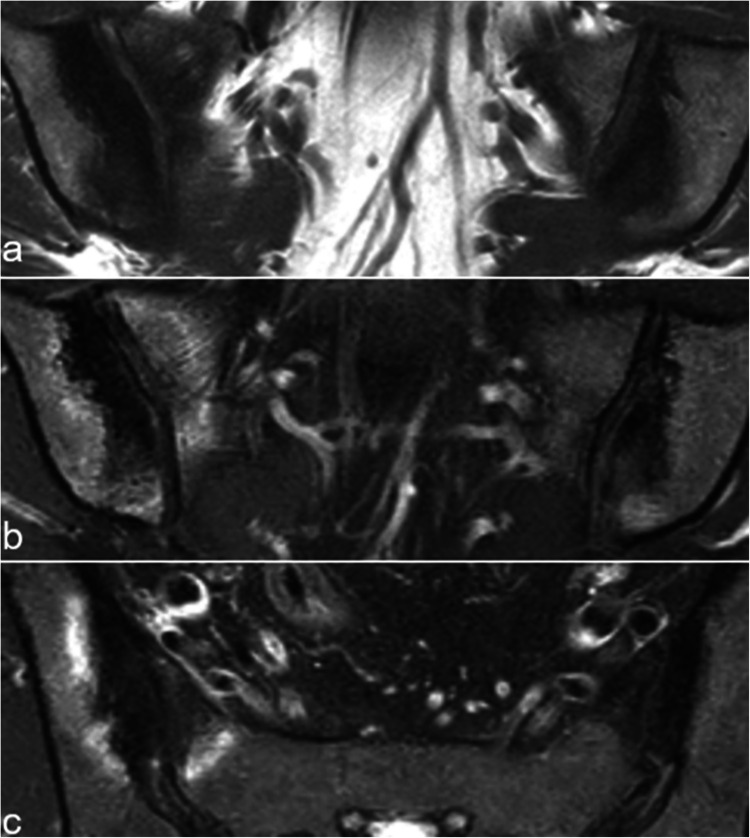
A 37-year-old woman with OCI. Coronal oblique **a** T1- and **b** FS T2-weighted images. **c** Axial oblique FS T2-weighted image show bilateral extensive hypointense subchondral sclerosis on the iliac side, which is well delineated, and surrounded by a mild rim of BME on the right side. Similar changes are observed on the sacral side. These findings predominate at the anterior aspect of the middle third of the SIJ

While radiographic and CT findings are typically diagnostic, some patients may be referred for MRI. OCI appears as a relatively homogeneous low signal intensity on all sequences, with well-defined margins, often surrounded by a mild rim of BME (Fig. 2) [11, 19, 21]. These iliac changes predominantly affect the anterior part of the middle third of the SIJ but usually extend beyond this region (Fig. 2) [11, 19, 20]. The extensive iliac subchondral sclerosis contrasts with the absence of adjacent erosions and the lack of involvement of the remaining SIJ, distinguishing OCI from sacroiliitis [19, 20]. Additionally, edematous, fatty or sclerotic changes may be observed on the adjacent sacral side of the joint (Fig. 2) [21].

**Fig. 3:**
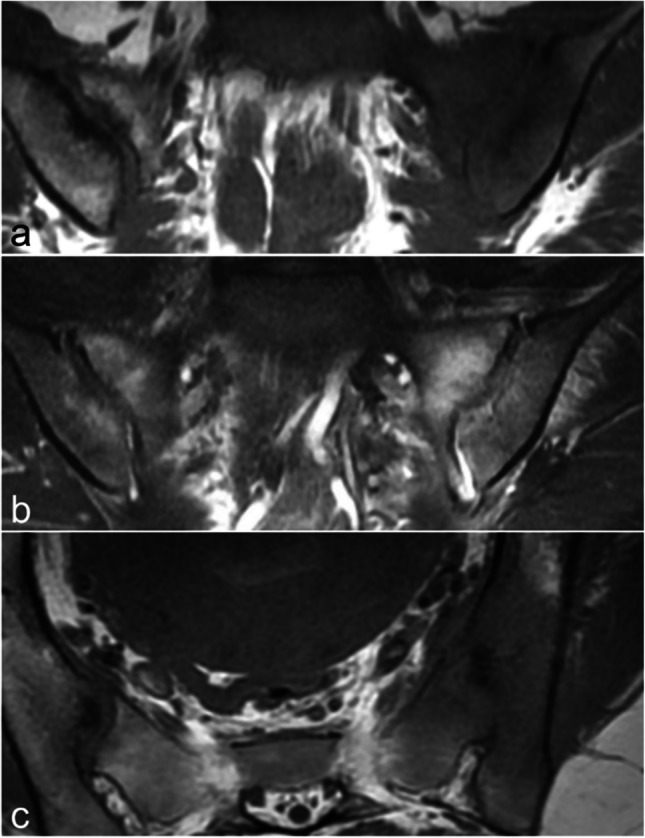
A 21-year-old woman, 9 days post-partum. Coronal oblique T1- (**a**) and T2 (**b**)-weighted images, axial oblique T1-weighted image (**c**). On the right side, mechanical changes related to pregnancy are observed, with subchondral sclerosis surrounded by mild BME; on the left side, there is extensive and pronounced subchondral BME with significant infiltration of the adjacent soft tissues, indicative of infectious sacroiliitis

Pregnancy-related subchondral BME of the SIJ is common in both pregnant and postpartum women, [18, 23–27]. A prevalence ranging from 27 to 73% has been reported during pregnancy [18, 23], peaking at 3 months postpartum [23] and gradually decreasing over time (12–41% at 12 months) [23, 25–28]. Patients may be either symptomatic or, more commonly, asymptomatic. Subchondral BME can sometimes be extensive, mimicking active sacroiliitis [2]. However, it typically affects the strain-related anterior aspect of the middle third of the SIJ, and significant erosive changes are rare (Fig. 3) [29]. When clear erosions are present, alternative etiologies such as infection or inflammatory spondyloarthritis should be considered, as further discussed in this manuscript. This BME can resolve, evolve into subchondral sclerosis, including OCI, or progress to areas with fat metaplasia [23, 26].

## Other strain related changes

Suchondral BME changes at the SIJ can also occur in healthy individuals [30], particularly in athletes and military recruits [31–33]. These changes are likely induced by mechanical strain [9]. They may involve the anterior part of the middle third of the SIJ, as previously described, or the superior or posterior lower ilium, probably reflecting a different distribution of mechanical forces [9, 11, 31–34]. They are usually limited in extent (seen on one or two contiguous images) and intensity compared to BME in axSpA, although they may rarely be more extensive [32–34]. Erosions are rare, but erosion-like irregularities and fat metaplasia have been reported in 13.6% of military recruits in one study [33]. Increased signal intensity in the ligamentous SIJ and along the iliac crest have also been exceptionally described on T2-weighted images [13, 17].

## Anatomical variants

**Fig. 4:**
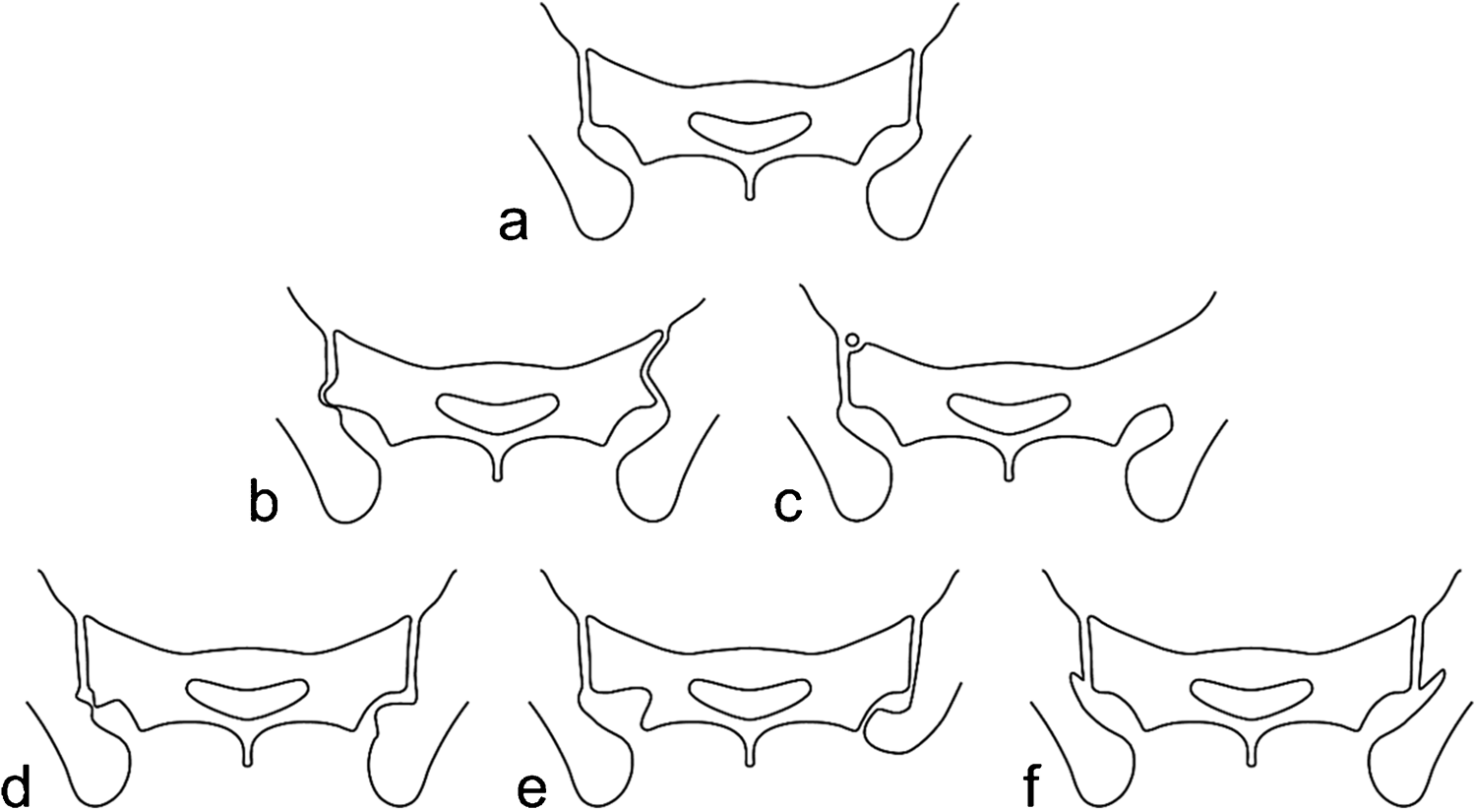
Figures of the anatomical variants of the SIJs on axial oblique images. **a** Normal shape, **b** dysmorphic joint affecting the posterior part of the joint on the right side and more diffuse on the left side, **c** unfused sacral ossification center on the right side and isolated synostosis on the left side, **d** accessory SIJ on the right side and iliosacral complex on the left side, **e** semicircular defect on the right side and crescent-like iliac bony plate on the left side, **f** bipartite iliac bony plate on both sides

SIJ variants can affect either the cartilaginous or ligamentous part of the joint (Fig. 4). Some of them are common, particularly in women, and may coexist within a single SIJ, raising the question of whether they represent normal anatomical features [22, 35–37]. These anatomical variants seem to occur more frequently in patients with mechanical low back pain [22, 38] and may increase the risk for erosions and BME in axial spondyloarthritis, possibly due to increased mechanical stress or altered physiological distribution within the joint [36, 39].

Optimal assessment of these variants is achieved through axial oblique MR images and CT-like sequences. They may be associated with misleading MR features such as subchondral BME and/or structural bone changes, or they may mimic enthesitis [37, 40].

### Variants involving the cartilaginous part of the SIJ

**Fig. 5:**
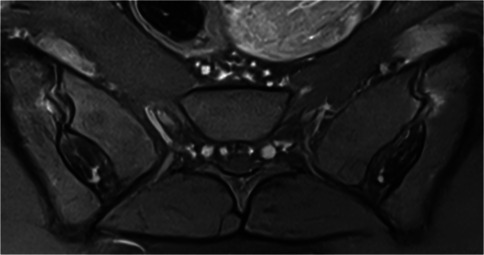
A 38-year-old woman with bilateral dysmorphic SIJ on axial oblique FS T2-weighted image: the central aspect of the joint is affected on the right side, the posterior aspect of the joint on the left side. Both are associated with subchondral edematous changes

Dysmorphic SIJ is defined by an abnormal shape of the cartilaginous joint due to osseous protrusions from either the sacrum or the ilium, with a corresponding groove in the opposite bone [22, 37, 41]. This variant was initially described at the posterior and distal aspect of the SIJ, where a convex cartilaginous sacral surface border protrudes into the adjacent iliac bone (Fig. 4) [37]. It can occur unilaterally or bilaterally. Recognizing dysmorphic SIJ is essential, as it can be associated with sclerotic and/or edematous changes occuring in an area not typically involved by mechanical stress and whose involvement should otherwise suggest sacroiliitis [37]. Axial oblique images provide a clearer analysis of this variant, which may be misleading on coronal oblique images. They demonstrate that the bone marrow changes are focal, limited in size, and centered by the dysmorphic joint at the distal and posterior part of the cartilaginous joint (Fig. 5) [11, 37]. Prominent cystic changes may also be observed. Other dysmorphic changes of the SIJ may be encountered in clinical practice, but their specific characteristics and impact on clinical and MRI features require further research (Fig. 5) [7, 41].

An unfused ossification center is a rare variant, even when evaluated using very thin slices or CT-like images (Fig. 4) [35]. When located at the anterosuperior aspect of the sacrum, it typically appears as a separate, often triangular bone structure and is usually asymptomatic. Persistence of unfused ossification centers in other parts of the joint has been reported, particularly between S1 and S2 where iliac involvement or atypical osseous fusion may also occur. In this latter location, BME and erosion-like lesions can mimic sacroiliitis, making differentiation from axSpA sacroiliitis challenging, especially in patients with low back pain [41].

Isolated synostosis is exceptionally rare and typically focal, unilateral and isolated, without any associated inflammatory or structural changes of the bone marrow adjacent to the synostosis or in the remaining or contralateral SIJ (Fig. 4) [37, 40, 42]. Recognizing this normal variant is essential to distinguish it from much more common acquired SIJ ankylosis resulting from ankylosing spondylitis or infectious sacroiliitis. Acquired ankylosis can also occur in association with SIJ hyperostosis.

### Variants involving the ligamentous part of the SIJ

**Fig. 6:**
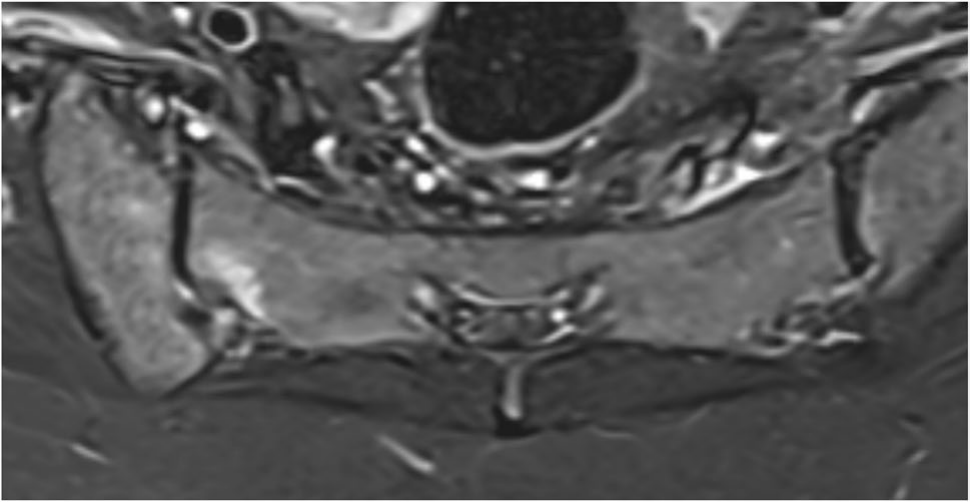
A 41-year-old woman with a right accessory SIJ showing edematous and sclerotic changes of the facing bones on axial oblique FS T2-weighted image

The accessory SIJ is a common variant with increasing prevalence with age [38]. This acquired pseudoarticulation develops secondary to altered biomechanical stresses, predominantly leading to fibrocartilaginous formation [38]. It is located dorsally within the ligamentous part of the joint, close to or at a distance from the cartilaginous joint (Fig. 4) [37, 41]. It is most frequently observed at the level of S2, just lateral to the dorsal sacral foramen, but it can also occur at S1, particularly in males [38, 41]. The opposing sacral and iliac facets may appear congruent, flat, slightly convex, or concave [9, 11, 40]. This variant can be unilateral or bilateral [38]. On MRI, the facing bones may exhibit edematous, sclerotic, fatty, and/or cystic changes due to degenerative or microtraumatic changes (Fig. 6). This variant may therefore be misinterpreted as sacroiliitis on coronal oblique images. However, it is well visualized on axial oblique images.

The iliosacral complex is defined as a prominent convex notch of the ilium corresponding to a concave depression of the posterolateral sacrum, without a visible joint (Fig. 4) [9]. This common variant is mostly bilateral and most often observed at the level of the first and second sacral foramina. Since this variation does not involve direct apposition of opposing bony surfaces, it is not associated with edematous or structural bony changes [11, 40]. However, prominent vessels within the reduced dorsal space may mimic enthesitis on oblique coronal T2-weighted images, as previously reported [37, 40].

The semicircular defect is a focal, prominently rounded concave depression of the upper aspect of the sacrum, above the level of the first foramen (Fig. 4). It is often bilateral and may, in rare cases, be associated with an opposing iliac defect. This variant does not exhibit adjacent significant BME or structural changes but may contain prominent vessels, which may mimic enthesitis on coronal oblique T2-weighted images [9, 37].

The crescent-like iliac bony plate is characterized by an anteriorly concave configuration of the iliac bone, with a relatively congruent bulging of the opposite sacral bone at the superior ligamentous joint compartment (Fig. 4). This variant is not associated with imaging pitfalls but appears to be more common in patients with mechanical back pain [13].

The bipartite iliac bony plate variant is defined by a deep indentation in the dorsal and distal part of the iliac bone, resulting from the deep insertion of a distal portion of the posterior sacroiliac ligament, often accompanied by surrounding vessels (Fig. 4) [6, 9]. This cleft-like appearance is typically bilateral and is best visualized on axial oblique images. On coronal oblique images, it appears as a channel-like appearance parallel to the SIJ, which may occasionally mimic edematous or erosive changes [9].

## Other normal features

### Pediatric SIJ

Assessing SIJ MRI in children and adolescents can be challenging due to normal developmental changes that occur in the immature skeleton [43]. In children, cartilaginous epiphyses and newly formed subchondral bone appear as areas of moderately increased signal on T2-weighted images, particularly on the sacral side of the joint. These normal, symmetrical MR features manifest as smooth symmetric linear bands with a mildly high T2 signal along both SIJs [44, 45]. This appearance varies with age and gender and typically extends along or partially along unfused sacral apophyses in most prepubertal children [44, 45]. It can be seen at the distal iliac side of the joint but is less common and less pronounced. Therefore, MRI features that should raise suspicion of sacroiliitis include an asymmetrical appearance, focal sacral involvement, intense high signal intensity on fat-suppressed T2-weighted images, greater intensity on the iliac side compared to the sacral side, and a large area of involvement [44, 45].

The presence of a small amount of fluid in the SIJ space can be considered a normal finding [46]. However, significant fluid accumulation or an asymmetrical distribution should raise suspicion of an underlying abnormality. In case of doubt, enhancement of the joint after gadolinium injection may help confirm inflammation.

**Fig. 7:**
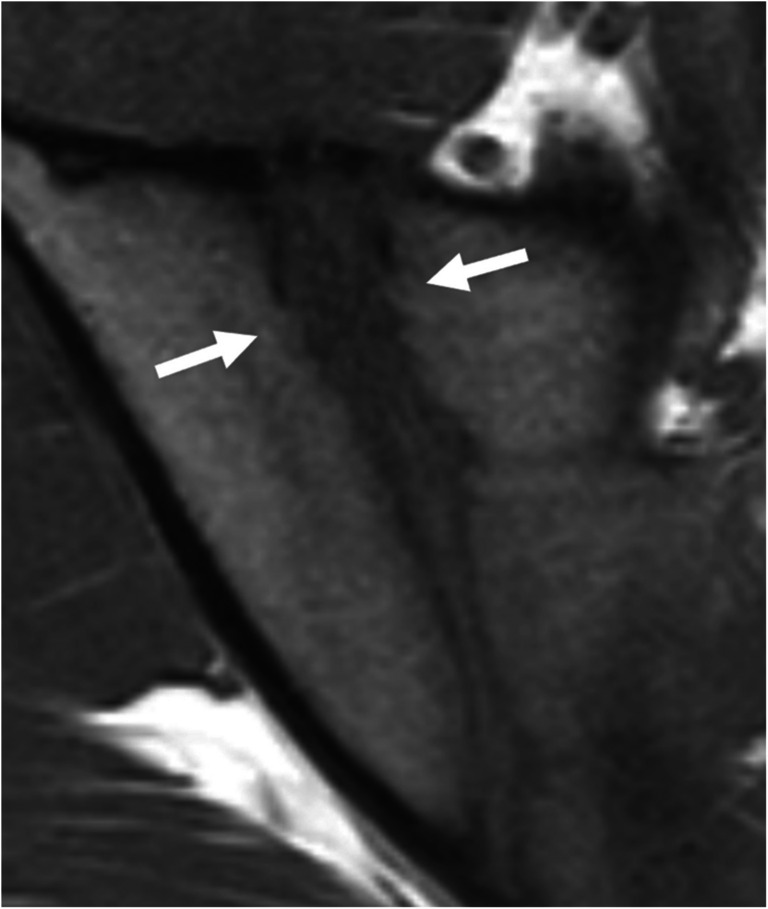
A 17-year-old man with irregularities of the SIJ mimicking erosions on coronal oblique T1-weighted image

Cortical bone irregularities, blurring, or undulations mimicking erosions are particularly frequent during the peripubertal phase, especially in the upper iliac quadrants (Fig. 7) [44, 46, 47]. However, unlike true erosions, they usually have smooth and regular margins and are not associated with adjacent sclerosis or fat infiltration.

### Vessels

Longitudinal vessels are abundant in the transitional cartilaginous-ligamentous portion of the SIJ. On coronal oblique images, the partial volume effect on these vessels may mimic either enthesitis or BME [11]. However, their tubular structure or rounded cross-sectional appearance is usually well demonstrated on axial oblique images, distinguishing them from the ill-defined edema characteristic of enthesitis in the ligamentous portion of the SIJ [11].

Additionally, the physiological regression of erythropoietic marrow with age can resemble fat metaplasia observed in axSpA [48, 49]. However, it presents with a patchy distribution and is unrelated to the SIJ, distinguishing it from the well-defined subchondral fat metaplasia seen in spondyloarthritis.

## Hyperostosis

**Fig. 8:**
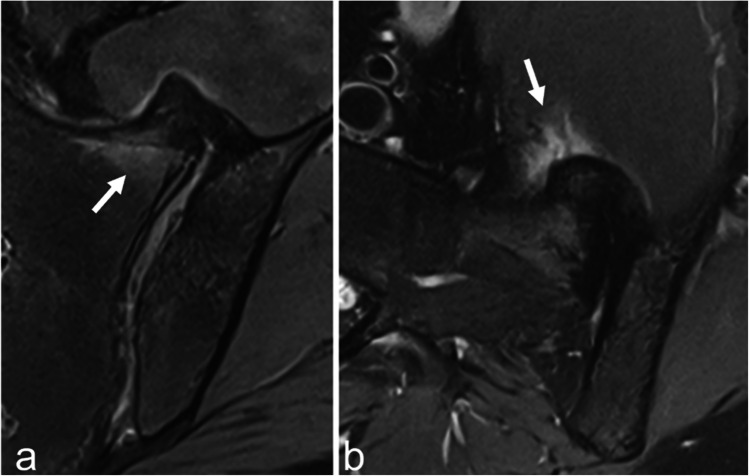
A 68-year-old man with hyperostosis of the SIJ. Slight BME at the base of the bridge (arrow) and in the adjacent soft tissues (arrow) on coronal oblique (**a**) and axial oblique (**b**) FS T2-weighted images

Diffuse idiopathic skeletal hyperostosis (DISH) is a common bone-forming disorder characterized by new bone formation in the axial and peripheral skeleton. While often asymptomatic, some patients may experience back pain and stiffness. In DISH, SIJ involvement manifests as extra-articular coarse bony bridges affecting the anterior, superior and posterior capsuloligamentous structures of the SIJ, sometimes accompanied by partial fusion in the upper part of the SIJ [50, 51]. Although mild BME may be observed at the basis of the bridges and sometimes in the adjacent soft tissues (Fig. 8), erosions and subchondral sclerosis are rare, helping to distinguish DISH-related changes from those seen in axSpA [52, 53].

## Infectious sacroiliitis

The clinical and biochemical manifestations of infectious sacroiliitis can vary, which may delay diagnosis. MRI changes can be detected within 3 days of symptom onset in pyogenic sacroiliitis [55]. Infectious sacroiliitis is typically unilateral. BME is usually intense (with pronounced low signal intensity on T1-weighted images) and extensive (Fig. 3). It is associated with significant joint fluid accumulation, bulging or ruptured inflamed capsuloligamentous structures, peri- and extra-articular soft tissue edema, and later on, soft tissue abscesses and erosive changes [11, 54–58]. Sclerosis, sequestrum, and calcifications within abscesses are more frequently observed in tuberculosis [54]. Intravenous gadolinium administration is essential for assessing this disease. Over time, bony bridges, fatty replacement, and ankylosis may develop.

## SAPHO syndrome

The synovitis, acne, pustulosis, hyperostosis, and osteitis (SAPHO) syndrome should be considered in the differential diagnosis of unilateral osteitis, typically confined to the sacrum or, more commonly, the iliac bone, with minimal involvement of the SIJ [59]. The MRI appearance varies depending on the stage of disease. BME is observed during the active phase, while hyperostosis, sclerosis, and fat metaplasia predominate in the chronic phase.

## Hyperparathyroidism

**Fig. 9:**
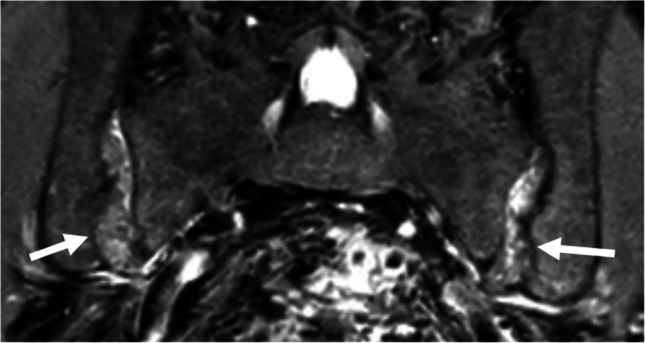
A 31-year-old man with chronic kidney disease. Extensive subchondral bone resorption on the iliac side of both SIJs (arrows) without adjacent BME or sclerosis on coronal FS T2-weighted image

Extensive subchondral bone resorption, predominantly or exclusively located on the iliac side of both SIJs, can occur in primary or secondary hyperparathyroidism, potentially mimicking sacroiliitis (Fig. 9). However, the large erosion-like lesions and huge pseudo-widening of the SIJ are unusual in inflammatory sacroiliitis [11, 60]. While adjacent BME and sclerosis may accompany bone resorption, the latter is more prominent, in contrast to axSpA [11]. Additionally, brown tumors can be observed, particularly in patients with chronic kidney disease.

## Sacral stress fractures

Sacral stress fractures can be classified into insufficiency and fatigue fractures [61].

**Fig. 10:**
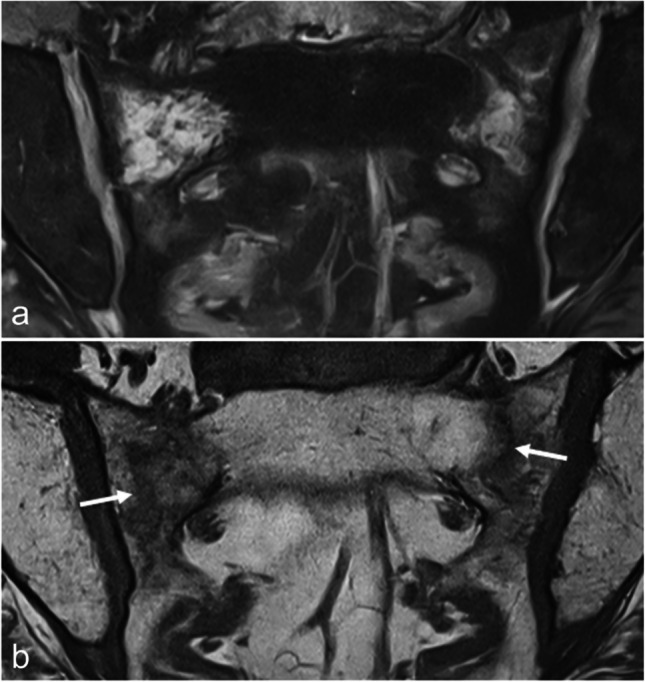
A 72-year-old woman with bilateral sacral insufficiency fractures. Sacral BME is observed on the coronal oblique FS T2-weighted image (**a**) whereas thin hypointense fracture lines (arrows) paralleling the SIJs are better demonstrated on the coronal oblique T1-weighted image (**b**)

Insufficiency fractures result from normal activity or minimal trauma on a sacrum that is deficient in microstructure and/or mineralization [9, 11, 61]. They are most commonly seen in osteoporotic middle-aged and elderly females or after radiotherapy, although younger patients can also be affected (e.g., those with anorexia nervosa or chronic inflammatory bowel diseases) [11, 62]. They are frequently bilateral and are typically vertical, paralleling the SIJ, often with a transverse component that resembles the letter “H”, hence the designation “Honda sign,” “H sign,” or “H pattern” [11]. On MRI, attention is drawn to a more or less extensive BME involving the sacral side of each SIJ (Fig. 10). Fractures can be identified as thin hypointense irregular lines, roughly paralleling the SIJ. While BME located to or predominantly involving the sacral side can occur in inflammatory sacroiliitis, the bilateral, relatively symmetrical distribution of the BME (sometimes with the H pattern), the presence of fracture lines, the absence of structural changes in the SIJ, and the patient’s age should raise suspicion for sacral insufficiency fractures [11]. Concomitant pelvic insufficiency fractures are common and should be carefully assessed [62].

Sacral fatigue fractures are less frequent than the insufficiency fractures. They occur in normal bone subjected to abnormal repetitive loads. These fractures are primarily seen in professional or collegiate athletes, as well as military recruits or police officers in training [63, 64]. However, lower bone mineral density can complicate the distinction between fatigue and insufficiency fractures, particularly in female athletes [63]. Fatigue fractures are typically unilateral and may present with an oblique pattern, extending from the superolateral aspect of the sacral wing to the first sacral foramen, although more vertical fractures may also be encountered. They are typically surrounded by BME.

## Other disorders

Malignancies such as metastases, myeloma, or lymphoma may rarely be misleading when bone marrow replacement involves the subchondral bone of the SIJ [11]. Crystal disorders such as gout and calcium pyrophosphate deposition disease rarely involve the SIJ but can mimic sacroiliitis, particularly during acute flares of the disease.

## Conclusion

In conclusion, even if subchondral changes such as BME are crucial for diagnosing sacroiliitis, they can also be observed in a variety of other conditions. Factors such as age, medical history (notably pregnancy), clinical context (including sport activity), the extent, intensity and topography of BME, concomitant structural changes, and whether the SIJ involvement is isolated or plurifocal, should all be considered. It is important to remember that non-inflammatory BME is more common than sacroiliitis [9, 11].

## Data Availability

Data illustrating this review are available from the corresponding author. The data are not publicly available due to privacy or ethical restrictions.
